# Supplementary material for: Co-appearance of superconductivity and ferromagnetism in a Ca2RuO4 nanofilm crystal
Source: Sci Rep. 2020 Feb 26;10:3462. doi: 10.1038/s41598-020-60313-x (PMC7044234; doi:10.1038/s41598-020-60313-x)
Supplement: Supplementary file 1 — Supplementary Information. [file 41598_2020_60313_MOESM1_ESM.pdf]

Supplementary Information for "Co-appearance of superconductivity and ferromagnetism in a Ca_2RuO_4 nanofilm crystal"

Hiroyoshi Nobukane,* Kosei Yanagihara, Yuji Kunisada, Yunito Ogasawara, Kakeru Isono, Kazushige Nomura, Keita Tanahashi, Takahiro Nomura, Tomohiro Akiyama, and Satoshi Tanda

*Corresponding author. Email: nobukane@sci.hokudai.ac.jp (H.N.)

Estimation of critical current density for thin film superconductors.

Here we discuss the estimation of the critical current density in more detail. According to Ref. [71], in a YBCO thin film, the critical current density is $\sim 10^5$ A/cm^2. Measured J_c values are summarised in Ref. [52]. We note that a high-T_c FeSe monolayer with an onset T_c above 40 K exhibited a large critical current density of $j_c \sim 1.7\times10^6$ A/cm^2 at 2 K [72], which is two orders of magnitude higher than $j_c \sim 2.2 \times 10^4$ A/cm^2 at 1.8 K for bulk FeSe with $T_c = 11$ K [73]. Therefore, the critical current density in the thin film is expected to be larger than for bulk crystal. Since the crystal structure of layered perovskite Ca_2RuO_4 is similar to that of a representative high-T_c superconducting material $La_{2-x}Sr_xCuO_4$, the critical current in Ca_2RuO_4 thin film may be also considered to be $j_c \geq 10^4$ A/cm^2. At $I_{\mathrm{QCP1}} = 100$ nA and $I_{\mathrm{QCP2}} = 1960$ nA in Fig. 3, the estimated current densities are $j \approx 8.3 \times 10^2$ A/cm^2 and $j \approx 1.6 \times 10^4$ A/cm^2, respectively. Sample 6 exhibits a supercurrent below $I_c = \pm200$ nA and $j_c = 1.6 \times 10^4$ A/cm^2 at 5 K (Fig. S2e). Table S1 summarizes the current densities obtained from our samples. We have observed the BKT behavior of $\alpha = 3$ at 5 K even in 17 T as shown in Fig. S2e. The critical magnetic field is expected to be larger than 17 T due to its short coherence length.

2D electric transport properties in Ca_2RuO_4 nanofilm crystals.

The dependence of $R_{\square/layer}$ on temperature is qualitatively different from the thermal activation type exponential temperature dependence in bulk Ca_2RuO_4. We found that the $R_{\square/layer}$ at 500 nA was characterized by a 2D variable-range-hopping (VRH), $R(T) =$

Sample No.	Thickness (nm)	j (A/cm^2) [at I (nA)]	Electronic state at 4.2 K
1	30	2.7×10^2 [100]<br>1.4×10^4 [5000]	SC
2	10	8.3×10^2 [(I_{QCP1} =)100]<br>1.6×10^4 [(I_{QCP2} =)1960]	SC
3	10	1.1×10^2 [13]	SC
4	12	6.9×10^2 [100]	SC
5	11	7.9×10^4 [4000]	SC
6	10	1.6×10^4 [200]	SC
7	50	1.7×10^2 [50]	Semi-metal
8	300	3.3×10^{-1} [1]	Insulator

Table S1. Summary of j and electronic states of Ca_2RuO_4

$R_0\exp(T_0/T)^{1/3}$, in the 10 – 65 K range, where T_0 becomes 1280 K as shown in Fig. S1. The result suggests the 2D conduction.

Figure S2a shows the dependence of the sheet resistance $R_{\square/layer} = \rho/d$ of sample 4 on temperature for different bias currents I, where the interlayer distance of Ca_2RuO_4 $d = 6.13$ Å , and $\rho = 2.0 \times 10^{-4}$ Ω·cm is the resistivity at 290 K. The resistivity of sample 4 is much smaller than that in the ab-plane $\rho_{ab} \sim 6$ Ω·cm in bulk Ca_2RuO_4 and is more like the resistivity under hydrostatic pressure above 0.5 GPa [29]. At a low bias current of $I = 20$ nA, the sheet resistance decreases to zero within the accuracy of our measurement. Although the onset temperature is rather high at 52 K, this broad transition behaviour occurs within a wide temperature range of 43 K. Figure S2b shows the $I - V$ characteristics of sample 4 for several selected temperatures. The result clearly shows supercurrents of $\sim \pm 100$ nA for the low bias current region, which strongly suggests the presence of superconductivity in a Ca_2RuO_4 flake.

The typical behaviour for a 2D superconductor is shown in Fig. S2. We analyse the broad transition based on the theory proposed by Berezinskii, Kosterlitz and Thouless [47, 48]. BKT theory predicts that a quasi-long-range order will be established in 2D systems below the BKT transition temperature T_{BKT}. Above T_{BKT}, the resistance resulting from

the free motion of vortices and antivortices is expressed as Halperin-Nelson equation [74], $R \propto R_N \exp\left[-2\left(b\frac{T_{c0}-T}{T-T_{\mathrm{BKT}}}\right)^{1/2}\right]$, where b is a constant of the order of unity, and the mean-field transition temperature T_{c0}. In contrast, below T_{BKT} a vortex and an antivortex form a pair. Here we determined T_{c0} by analyzing the resistance data through the 2D Aslamasov-Larkin (AL) model [75], $\frac{R_N}{R_\square} - 1 = \frac{e^2}{16\hbar} R_N \left(\frac{T_{c0}}{T-T_{c0}}\right)$, where R_N is the normal sheet resistance. The sheet resistance at T_c^{onset} is used as R_N. The values of T_{c0} at low bias current are found to be 70.4 and 23 K in samples 1 and 4, respectively in Figs. S2a and S2b). The fitting parameter T_{BKT} is 9 K for $I = 20$ nA in sample 4. The fitting results are shown in the inset of Fig. S2a. The theoretical expression of the resistance fits nicely with our experimental data. Another important feature of the BKT transition is the presence of a universal jump in the $I - V$ characteristics $V \sim I^\alpha$ owing to the current-induced dissociation of vortex-antivortex pairs. The exponent α jumps abruptly from 1 to 3 with decreasing temperature, and this has been reported in various 2D superconducting systems such as a Hg-Xe alloy [76], an ultrathin $YBa_2Cu_3O_{7-\delta}$ film [44], and a superconducting wire network [77]. The $d\log(V)/d\log(I)$ plot shows that α jumps from 1 to more than 3 at a low temperature as shown in the upper and lower insets of Fig. S2b. The plot are well fitted at three orders of magnitude. T_{BKT} is estimated to be 9.5 K, which is consistent with $T_{\mathrm{BKT}} = 9$ K determined from the resistance data at $I = 20$ nA for sample 4 in Fig. S2a. We have also confirmed the BKT transition in different Ca_2RuO_4 thin films as shown in Fig. S2e. On the basis of the above standard analysis, we suggest that a Ca_2RuO_4 thin film shows a typical BKT transition for a 2D superconductor, namely a topological phase transition. Recently, however, a role of superconducting inhomogeneity in nonlinear $I - V$ characteristics of 2D superconductors has been reported in $SrTiO_3$ interfaces [78]. The inhomogeneity is important in our results. So, we need to investigate further the relationship between the BKT physics and emrgent inhomogeneity in a layered Ca_2RuO_4.

Difference of transport properties in clean and dirty systems.

Samples 2 and 4 have approximately the same thickness at 10 and 12 nm, respectively. The resistivity for sample 2 exceeds that of sample 4 at 280 K. This means that the sample 2 contains more disorder or intrinsic inhomogeneity than sample 4. Sample 4 showed the insulating behavior at 500 nA. We found that the resistance was characterized by a 2D VRH in the $10 - 65$ K range, where T_0 becomes 1280 K. In the “clean” system (sample 4),

the current-induced superconductor-“Mott insulator” transition occurs. In sample 2 with the strong disorder or inhomogeneity, the small ferromagnetic superconducting domains can be locally developed at high temperatures, while clean samples begin to grow larger superconducting correlations in the sample at lower temperatures. This situation can also be explained from observation of the hysteresis of the $I - V$ curve in the superconducting state and the critical exponent of $z\nu = 1.5$ in the “disordered” SI transition. The clean and dirty samples show different universality classes. Indeed, there are fluctuations in the superconducting amplitude and phase, which give the broad transition. Thus, the difference of the transport behavior depends on the strength of disorder or inhomogeneity in the samples with the same thickness. This may affect the difference of T_c. In the future, we need to clarify the difference of T_c in both clean and dirty systems.

Sample	Thickness (nm)	ρ(280K) ($\Omega\cdot$ cm)	T_c^{onset}(K)	T_{BKT}(K)	Hysteresis of IV	$z\nu$		SC domains
2	10	1.3×10^{-2}	80	16	○	0.64 (High-T)	1.5 (Low-T)	small
4	12	2.4×10^{-4}	52	9	×	0.68		large

Table S2. Comparison of samples 2 and 4.

Resistance anomalies around 100 and 200 K.

Fig. S6 shows the temperature dependence of $R_{\square/layer}(T)/R(290\text{K})$ for different thicknesses. Interestingly, we observed resistance anomalies around 100 and 200 K. In bulk crystals, near 200 K, A-centered antiferromagnetic (AFM) ordering at ~ 170 K, B-centered AFM ordering at ~ 140 K [32] and orbital ordering at ~ 260 K [79] have been reported. The resistance anomalies at ~ 100 and ~ 180 K were detected in sample 7, which becomes an insulator. On the other hand, around ~ 100 K, the onset temperature T_c^{onset} was observed in nanofilms. We have observed the ferromagnetic order below 180 K through the magnetic measurement. The ferromagnetic transition is related to the resistance anomaly in the vicinity of 200 K for nanofilms (samples 1 and 2). Moreover, as shown in Fig. S6, a precursor superconducting phenomenon may be observed at a rather high temperature $T^* = 215$ K in sample 1 because the bias-current dependence of the resistance appears below T^*. In Ref. [34], the magnetism under 5 mA begins to decrease below 220 K. This is close to $T^* = 215$ K observed in sample 1. In addition to these results, according to arguments related to the pseudogap in cuprate superconductors, $T \sim 200$ K may indicate the temperature for preforming Cooper pairs. The resistance behavior at 3000 nA for sample 1 is similar to the behavior of a ferromagnetic metal under pressure in bulk [29].

First-principles calculations.

We performed first-principles calculations based on spin-polarized density function theory, using a local spin-density approximation (LSDA), which is implemented in the plane-wave and projector augmented wave method the Vienna Ab-initio Simulation Package (VASP 5.4.1) [80–85]. We adopted the LSDA+U scheme [86] with a U_{eff} of 2.5 eV, which has been reported to reproduce the band gap of bulk Ca_2RuO_4 [87]. We used a supercell consisting of 28 atoms for a bulk system. We also considered a 1×1 monolayer, a bilayer and a trilayer of Ca_2RuO_4 with a 15 Å vacuum layer to model a thin film. We applied a 500 eV cutoff for the plane-wave basis set and a Gaussian smearing model of $\sigma = 0.05$ eV. $4 \times 4 \times 2$ and $4 \times 4 \times 2$ Monkhorst-Pack special k-point grids [88] for the first Brillouin zone sampling were used, respectively, for a bulk system and a slab model. All the atoms were relaxed until the force on each atom was less than 0.02 eV/Å.

We also performed the first-principles calculation with simple LSDA and HSE06 hybrid functionals. The results with these functionals also show the same trends, such as

a decreasing tilting angle, through monolayer exfoliation. In addition, LSDA+U provides similar results to those obtained with hybrid HSE06 functionals. These points validate the LSDA+U methods. Table S3 summarizes the result of the first-principles calculations.

		a(Å)	b(Å)	c(Å)	tilting(°)
LDA	Monolayer	5.546	4.777	-	6.27
	Bulk	5.318	5.201	12.042	7.90
LDA+U	Monolayer	5.806	4.847	-	10.01
	Bulk	5.592	5.253	11.548	11.65
HSE06	Monolayer	5.693	5.127	-	10.65
	Bulk	5.700	5.375	11.648	11.95

Table S3. Summary of the first-principles calculations for Ca_2RuO_4.

[71] Mogro-Campero, A., Turner, L. G., Hall, E. L., Garbauskas, M. F. & Lewis, N. Epitaxial growth and critical current density of thin films of $YBa_2Cu_3O_{7-x}$ on $LaAlO_3$ substrates. *Appl. Phys. Lett.* **54**, 2719 (1989).

[72] Zhang, W. H. *et al.* Direct observation of high-temperature superconductivity in one-unit-cell FeSe films. *Chin. Phys. Lett.* **31** 017401 (2014).

[73] Lei, H., Hu, R. & Petrovic, C. Critical fields, thermally activated transport, and critical current density of β-FeSe single crystals. *Int. J. of Mod. Phys. B* **24** 4081 (2010).

[74] Halperin B. I., Nelson, D. R. Resistive transition in superconducting films. *J. Low. Temp. Phys.* **36** 599 (1979).

[75] Aslamazov, L. G. & Larkin, A. I. The influence of fluctuation pairing of electrons on the conductivity of normal metal. *Phys. Lett.* **26A**, 238 (1968).

[76] Kadin, A. M., Epstein, K. & Goldman, A. M. Renormalization and the Kosterlitz-Thouless transition in a two-dimensional superconductor. *Phys. Rev. B* **27**, 6691 (1983).

[77] van der Zant, H. S. J., Webster, M. N., Romijn, J., & Mooij, J. E. Vortices in two-dimensional superconducting weakly coupled wire networks. *Phys. Rev. B* **50**, 340 (1994).

[78] Venditti, G. *et al.* Nonlinear $I\Gamma V$ characteristics of two-dimensional superconductors: Berezinskii-Kosterlitz-Thouless physics versus inhomogeneity. *Phys. Rev. B* **100**, 064506 (2019).

[79] Zegkinoglou, I. *et al.* Orbital ordering transition in Ca_2RuO_4 observed with resonant X-ray diffraction. *Phys. Rev. Lett.* **95** 136401 (2005).

[80] Kresse, G. & Hafner, J. Ab initio molecular dynamics for liquid metals. *Phys. Rev. B* **47** 558(R) (1993).

[81] Kresse, G. & Hafner, J. Ab initio molecular-dynamics simulation of the liquid-metal amorphous-semiconductor transition in germanium. *Phys. Rev. B* **49** 14251 (1994).

[82] Kresse, G. & Furthmüller, J. Efficiency of ab-initio total energy calculations for metals and semiconductors using a plane-wave basis set. *Comput. Mater. Sci.* **6** 15 (1996).

[83] Blöchl, P.E. Projector augmented-wave method. *Phys. Rev. B* **50** 17953 (1994).

[84] Kresse, G., & Furthmüller, J. Efficient iterative schemes for ab initio total-energy calculations using a plane-wave basis set. *Phys. Rev. B* **54** 11169 (1996).

[85] Kresse, G. & Joubert, D. From ultrasoft pseudopotentials to the projector augmented-wave method. *Phys. Rev. B* **59** 1758 (1999).

[86] Dudarev, S.L., Botton, G.A., Savrasov, S.Y., Humphreys, C.J. & Sutton, A.P. Electron-energy-loss spectra and the structural stability of nickel oxide: An LSDA+U study. *Phys. Rev. B* **57** 1505 (1998).

[87] Fang, Z., Nagaosa, N. & Terakura, K. Orbital-dependence phase control in $Ca_{2-x}Sr_xRuO_4$. *Phys. Rev. B* **69**, 045116 (2004).

[88] Monkhorst, H.J. & Pack, J.D. Special points for Brillouin-zone integrations. *Phys. Rev. B* **13** 5188 (1976).

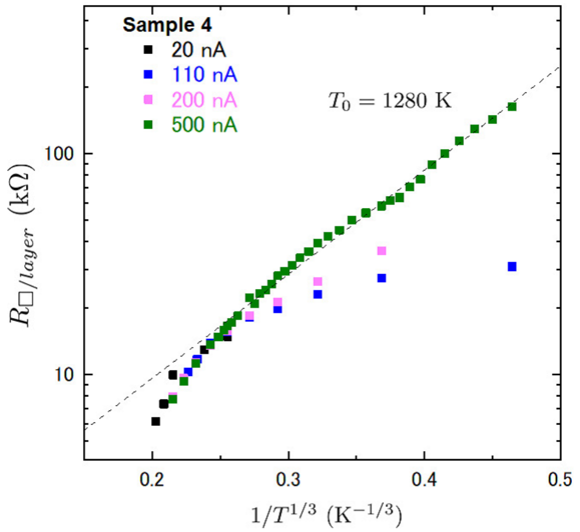

Figure S1. Sheet resistance as a function of $T^{-1/3}$ for bias currents in sample 4. The resistance at 500 nA fit to a linear line from 10 to 65 K, suggesting 2D VRH conduction.

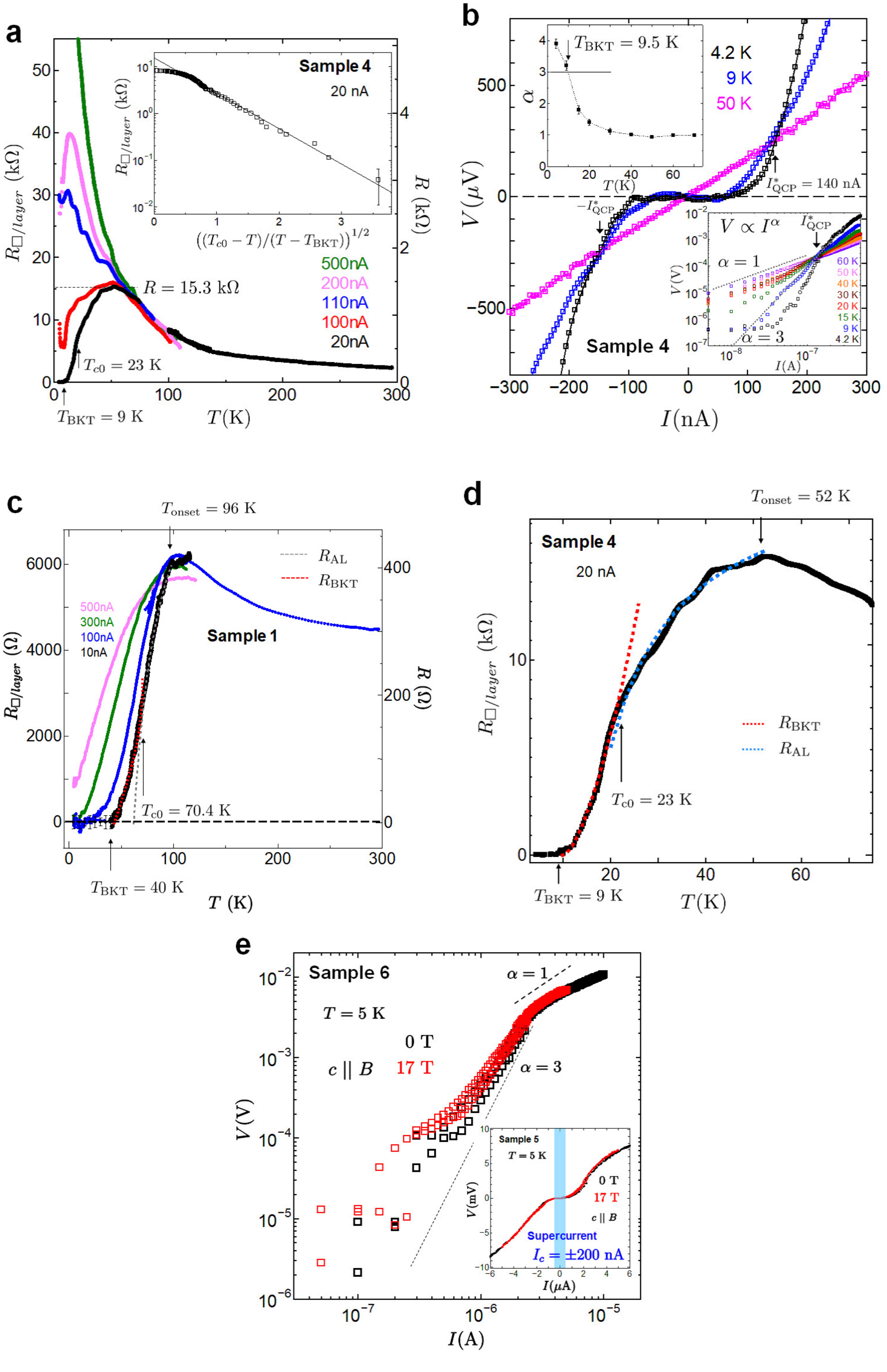

a
Sample 4
20 nA
$((T_{c0}-T)/(T-T_{\rm BKT}))^{1/2}$
$R_{\square/layer}$ (kΩ)
R (kΩ)
500nA
200nA
110nA
100nA
20nA
$R = 15.3$ kΩ
$T_{c0} = 23$ K
$T_{\rm BKT} = 9$ K
T(K)
b
$T_{\rm BKT} = 9.5$ K
α
T(K)
4.2 K
9 K
50 K
$V\,(\mu V)$
$I^*_{\rm QCP} = 140$ nA
$-I^*_{\rm QCP}$
$V \propto I^{\alpha}$
$\alpha = 1$
$\alpha = 3$
$I(A)$
$V(V)$
Sample 4
I(nA)
c
$T_{\rm onset} = 96$ K
$R_{\rm AL}$
$R_{\rm BKT}$
500nA
300nA
100nA
10nA
Sample 1
$R_{\square/layer}$ (Ω)
R (Ω)
$T_{c0} = 70.4$ K
$T_{\rm BKT} = 40$ K
T (K)
d
$T_{\rm onset} = 52$ K
Sample 4
20 nA
$R_{\square/layer}$ (kΩ)
$R_{\rm BKT}$
$R_{\rm AL}$
$T_{c0} = 23$ K
$T_{\rm BKT} = 9$ K
T(K)
e
Sample 6
$T = 5$ K
0 T
17 T
$c \parallel B$
$\alpha = 1$
$\alpha = 3$
V(V)
I(A)
Sample 5
$T = 5$ K
0 T
17 T
$c \parallel B$
Supercurrent
$I_c = \pm 200$ nA
V(mV)
$I(\mu A)$

Figure S2. **2D electric transport properties in Ca_2RuO_4 thin film crystals.** (**a**)Temperature dependence of the sheet resistance $R_{\square/layer}$ for various applied currents in sample 4 with a thickness of 12 nm. $R_{\square/layer}$ is resistance per square per RuO_2 layer. The inset shows the sheet resistance $R_{\square/layer}$ as a function of $[(T_{c0} - T)/(T - T_{\mathrm{BKT}})]^{1/2}$ at $I = 20$ nA. (**b**)$I - V$ characteristics at various temperatures in sample 4. The $I - V$ curves show supercurrent at 4.2 and 9 K. The dotted lines in the lower inset represent $V \sim I^3$ and $V \sim I$ dependence, respectively. The upper inset shows the temperature dependence of the exponent α extracted from power-law fitting $V \sim I^{\alpha}$. (**c**)Temperature dependence of sheet resistance and measured resistance in sample 1 for various bias currents. The grey dotted line represents the superconducting amplitude fluctuation taking into account the 2D Aslamazov-Larkin (AL) model, which give the mean-field transition temperature T_{c0}. We use the sheet resistance at T_c^{onset} as the R_N. The red dotted line represents the BKT transition using the Halperin-Nelson equation. (**d**)Temperature dependence of sheet resistance in sample 4 at 20 nA. The red and blue dotted lines represent the BKT and AL fitting curves, respectively. (**e**)$d\log(V)/d\log(I)$ and $I - V$ plots at 5 K in sample 6 when a magnetic field is applied parallel to the c axis. A supercurrent of ± 200 nA was observed in a zero magnetic field. The $I - V$ curves show no hysteresis.

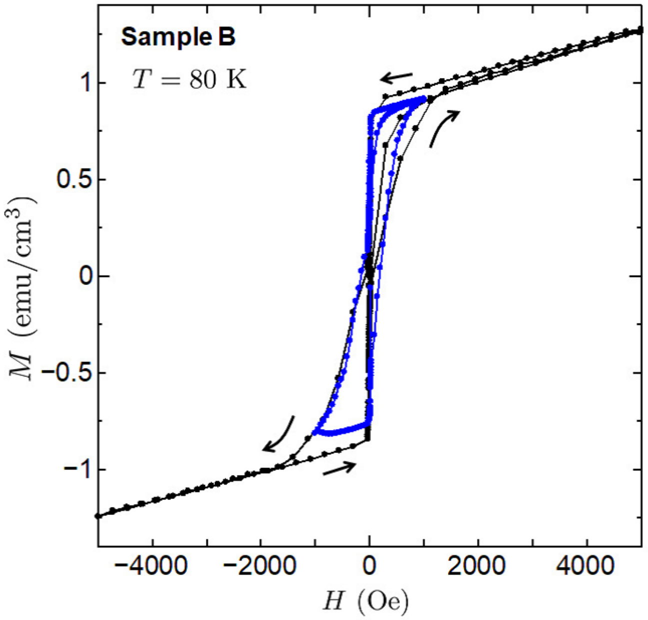

Figure S3. Magnetization as a function of magnetic field at 80 K in sample B. The non-saturating behavior in high-magnetic fields reveals the weak itinerant ferromagnetism of Ca_2RuO_4 nanofilms.

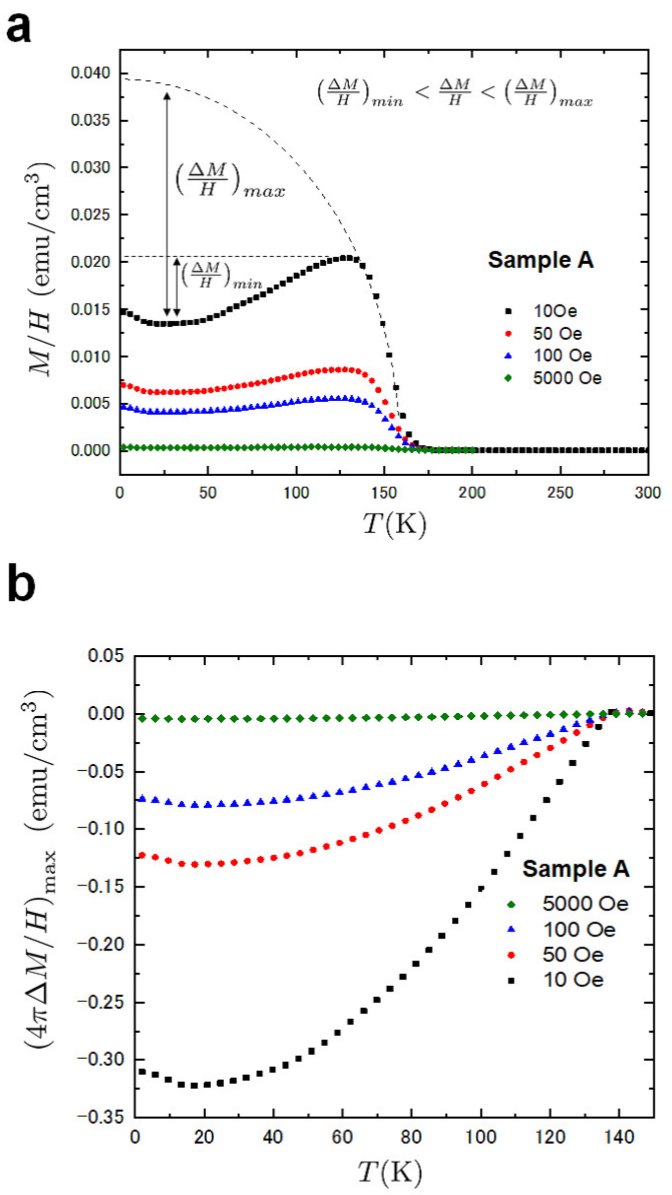

Figure S4. Analysis of $M - T$ curves for sample A by itinerant ferromagnetic fitting. (a)Dependence of magnetic susceptibility on temperature for the typical FC process for various magnetic fields. For the data in Fig. 2a, we fitted the temperature dependence of the magnetization with $M = M_0[1 - (T/T_{\rm Curie})^2]^{1/2}$ in the itinerant ferromagnetic systems [14, 49]. We can isolate the diamagnetic components by subtracting the fit from the measured points in various magnetic fields. (b)Temperature dependence of the diamagnetization for each field. In this analysis, the volume fraction of the superconductivity is $(4\pi\Delta M/H)_{\rm max} = -0.32$ emu/cm^3 at 10 Oe.

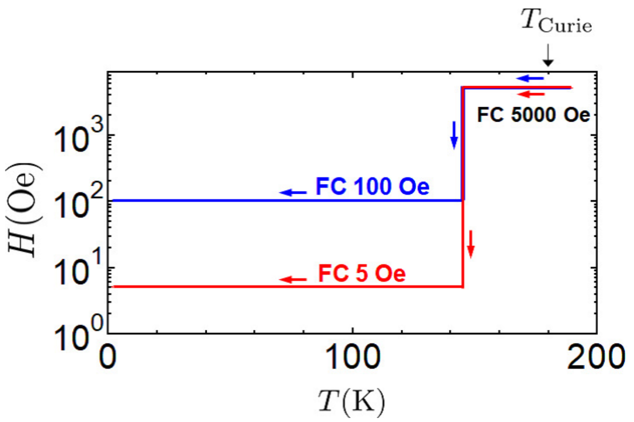

Figure S5. Field-cooled process of magnetic measurement for sample B to realize a ferromagnetic order. The sample was cooled from 190 to 145 K while maintaining a magnetic field of 5000 Oe. After waiting for 5 minutes at 145 K, we swept the magnetic field from 5000 Oe to fields of 5, 8, 10, 12 , 15, 100 Oe. And then, the dependence of magnetization M on temperature T was measured from 145 to 2 K. Fig. S5 shows the measurement process for 5 and 100 Oe. When measuring $M-T$ curves for various magnetic fields, the temperature was increased to 190 K ($> T_{\mathrm{Curie}}$) each time, and the FC process was carried out. The results for this FC process are shown in Figs. 2e and 2f.

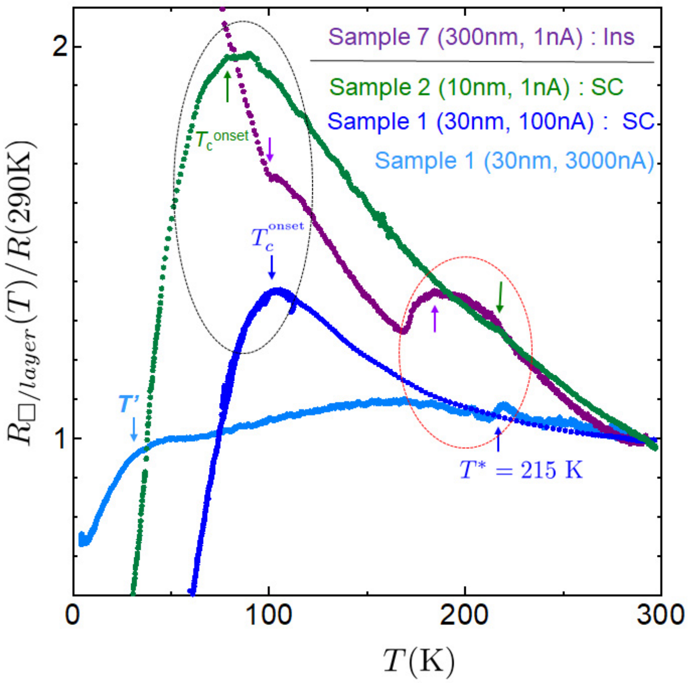

Figure S6. Resistance anomalies around 100 and 200 K. Temperature dependence of normalized sheet resistance $R_{\square/layer}(T)/R(290\mathrm{K})$ for different thicknesses of nanoscale Ca_2RuO_4. The resistance anomaly is reproduced in our samples. Around 100 K, the T_c^{onset} was observed in nanofilms. For nanofilms of samples 1 and 2, the resistance anomaly around 200 K is related to the transition to ferromagnetism and the preforming Cooper pairs (see Supplemental Text). In sample 1, bias-current dependence of the resistance appears below $T^* = 215$ K. The resistance behavior at 3000 nA for sample 1 is similar to the behavior of a ferromagnetic metal under pressure in bulk Ca_2RuO_4 [28].
